# Comparison of DAPT and Thrombolysis in Minor Stroke with Regard to Efficacy: A Meta-Analysis

**Published:** 2026-06-05

**Authors:** MUHAMMAD SHOAIB, HASEEB AHMED, AREEB JEHANGIR CHAUDHARY, ABIN ROY, WAFAA DRAO, RIDA-E ZAHRA, ZORAIZ HAIDER, MUHAMMAD SHAMS UL HAQ, ALIZAY FATIMA, SYED HAIDER FAROOQ SHERAZI, AHMED INTAKHAB

**Affiliations:** 1 Department of Cardiology, Chaudhary Pervaiz Elahi Institute of Cardiology, Multan, Pakistan; 1 Department of Cardiology, Chaudhary Pervaiz Elahi Institute of Cardiology, Multan, Pakistan; 2 Department of Surgery & Medicine, Combined Military Hospital (CMH), Rawalakot, Pakistan; 2 Department of Surgery & Medicine, Combined Military Hospital (CMH), Rawalakot, Pakistan; 3 Department of General Medicine, Fatima Memorial Hospital College of Medicine and Dentistry, Lahore, Pakistan; 3 Department of General Medicine, Fatima Memorial Hospital College of Medicine and Dentistry, Lahore, Pakistan; 4 Department of Medicine, Government Medical College, Kozhikode, India; 4 Department of Medicine, Government Medical College, Kozhikode, India; 5 Department of Medicine, University of Georgia, Tbilisi, Georgia; 5 Department of Medicine, University of Georgia, Tbilisi, Georgia; 6 Department of Neurology, Punjab Institute of Neurosciences, Lahore, Pakistan; 6 Department of Neurology, Punjab Institute of Neurosciences, Lahore, Pakistan; 7 Department of Emergency Medicine, Ahmad Hospital, Lahore, Pakistan; 7 Department of Emergency Medicine, Ahmad Hospital, Lahore, Pakistan; 8 Department of Internal Medicine, Midlands Regional Hospital, Tullamore, Ireland; 8 Department of Internal Medicine, Midlands Regional Hospital, Tullamore, Ireland; 9 Department of Ear, Nose & Throat (ENT), Ghurki Hospital, Lahore, Pakistan; 9 Department of Ear, Nose & Throat (ENT), Ghurki Hospital, Lahore, Pakistan; 10 Department of General Medicine, Medical Teaching Institution (MTI) Hayatabad Medical Complex, Peshawar, Pakistan; 10 Department of General Medicine, Medical Teaching Institution (MTI) Hayatabad Medical Complex, Peshawar, Pakistan; 11 Department of Medicine, Rawal General & Dental Hospital, Islamabad, Pakistan; 11 Department of Medicine, Rawal General & Dental Hospital, Islamabad, Pakistan

**Keywords:** minor ischemic stroke, dual antiplatelet therapy (DAPT), intravenous thrombolysis (IVT), symptomatic intracranial hemorrhage, functional outcomes, meta-analysis

## Abstract

**Objective:**

Minor ischemic stroke (NIHSS ≤ 5) presents a therapeutic dilemma, balancing the potential benefits of intravenous thrombolysis (IVT) against its hemorrhagic risks, with dual antiplatelet therapy (DAPT) established for recurrence prevention. This meta-analysis compares the efficacy and safety of dual antiplatelet therapy (DAPT) versus intravenous thrombolysis (IVT) in patients with minor stroke, examining functional recovery, the risk of symptomatic intracranial haemorrhage (SICH) and mortality.

**Methods:**

A systematic search of databases was done in PubMed, Embase, Scopus, and Cochrane until June 2025. The studies comparing DAPT and IVT were conducted in patients with minor stroke. Risk ratios (RR) at 95% confidence interval (CI) were pooled with a random-effects model. Heterogeneity was assessed using I2 statistics, and analyses were conducted using RevMan 5.4.

**Results:**

There was no substantial difference between DAPT and IVT in functional recovery i.e., mRS 0-1 (RR, 1.03; 95% CI 0.98-1.09; p = 0.24), mRS 0-2 (RR 1.00; 95% CI 0.98-1.03; p = 0.87). The risk of SICH was significantly lower when using DAPT (RR 0.10; 95% CI 0.03-0.29; p < 0.0001). There was a reduction in mortality that was not statistically significant with DAPT. The stability of results was established by sensitivity analysis.

**Conclusions:**

In minor ischemic stroke, DAPT provides similar functional recovery to IVT while offering a significantly improved safety profile through reduced SICH risk. DAPT may represent a safer alternative to thrombolysis in this patient population.

## Introduction

Minor ischemic stroke, commonly described by a National Institutes of Health Stroke Scale (NIHSS) score of ≤ 5, represents a substantial clinical challenge in cerebrovascular medicine due to its frequency and potential for disabling outcomes^1)^. Despite often mild initial neurological deficits, minor ischemic stroke is associated with a meaningful risk of long-term disability, recurrent stroke, and early neurological deterioration. Because of its prevalence and potential for incapacitating consequences, minor ischemic stroke─generally defined by an NIHSS score of ≤ 5─represents a significant therapeutic problem in cerebrovascular medicine^1)^. Globally, stroke ranks second among causes of death and third among contributors to disability. Stroke imposes a substantial economic burden; by 2050, its anticipated annual global cost is expected to nearly triple to around $890 billion, or 0.66% of global GDP. Approximately 65.3% of strokes are ischemic in nature, with intracerebral haemorrhage (28.8%) and subarachnoid haemorrhage (5.7%) making up the remaining percentages^2)^. More than 11.9 million stroke events occur annually worldwide, accounting for approximately 7.3 million deaths and 160.5 million disability-adjusted life years (DALYs). Although the initial symptoms may appear mild, over 30% of these people experience poor functional results, including significant disability, repeated stroke, or early brain deterioration^3)^. More than 11.9 million stroke episodes occur annually worldwide, attributed to 7.3 million deaths and 160.5 million disability- adjusted life years (DALYs), according to current estimates. Stroke remains a staggering global burden^4)^.

Clinical professionals face a therapeutic conundrum when managing small strokes, weighing the possible advantages of vigorous intervention against the dangers of treatment-related side effects. Dual antiplatelet treatment (DAPT) and intravenous thrombolysis (IVT) are the two primary therapeutic philosophies that now dominate the field^3)^. IVT, primarily using alteplase or tenecteplase, is guideline-recommended for acute ischemic stroke presenting within 4.5 hours of symptom onset; however, its role in minor stroke remains controversial, with conflicting evidence regarding functional benefit and bleeding risk^3, 4)^. Intravenous treatment (IVT), mostly with alteplase or tenecteplase, is advised in cases of acute ischemic stroke addressed within 4.5 hours of the initial presentation of symptoms; however, its effectiveness for mild stroke remains controversial due to conflicting findings^3, 4)^.

DAPT, aspirin combined with a P2Y12 inhibitor such as clopidogrel, is a viable alternative; however, according to research demonstrating lower rates of recurrent stroke without noticeably increased bleeding risk in high-risk transient ischemic attack (TIA) and mild stroke^2)^. Landmark trials in high-risk transient ischemic attack (TIA) and minor stroke populations have demonstrated reduced recurrent stroke rates without a significant increase in hemorrhagic complications, During the initial 4.5 hours of the beginning of symptoms onset, intravenous thrombolysis, primarily with alteplase or tenecteplase, is advised by guidelines for acute ischemic stroke. Its effectiveness in treating minor strokes remains debatable. There is an increased likelihood of SICH, with no appreciable benefit over antiplatelet treatment, despite some data suggesting that IVT improves functional outcomes^5, 6)^.

In contrast, DAPT, combining aspirin and clopidogrel, has emerged as a promising alternative based on trials like CHANCE and POINT, which demonstrated a lower incidence of recurrent stroke without increased hemorrhage risk in high-risk TIA and mild stroke^7)^.

The present meta-analysis seeks to resolve the ongoing therapeutic dilemma in the management of minor stroke by comprehensively assessing the efficacy and safety of DAPT versus IVT. It synthesises evidence on outnumbered females, with 4,151 males, including safety endpoints such as symptomatic intracranial hemorrhage. The findings aim to provide clinicians with robust evidence to inform individualised treatment decisions and guide future clinical guidelines. By synthesizing available evidence from direct-comparison studies, including functional outcomes and safety endpoints such as SICH and mortality, this analysis aims to inform individualized clinical decision-making and contribute to evidence-based guideline development.

## Study Design and Methods

The present systematic review and meta-analysis were performed according to the PRISMA (Preferred Reporting Items for Systematic Reviews and Meta- Analyses) guidelines, and the review protocol was registered with PROSPERO (CRD420251142882)^8)^.

### Search strategy

We searched PubMed, The Cochrane Library, and ScienceDirect for related articles using specific keywords combined with Boolean operators. The search strategy for each data base is listed as; "Pubmed (((((((minor stroke) OR (mild stroke)) AND (thrombolysis)) OR (IVT)) OR (t-pa)) OR (alteplase)) AND (dual antiplatelet)) OR (DAPT)”, “The Cochrane Library: (Minor stroke): ti, ab,kw AND (Thrombolysis): ti, ab,kw OR (alteplase): ti, ab,kw AND (DAPT): ti, ab,kw OR (dual antiplatelet therapy): ti, ab,kw”, “Science Direct: ((minor stroke) AND ((thrombolysis) OR (alteplase)) AND ((dual antiplatelet) OR (DAPT)))”. Articles were searched using filters to display only RCTs and cohorts published in the last 10 years, thereby including the most up-to-date data.

### Selection criteria

Articles were screened against a specific eligibility criterion, which included studies if they were RCTs or cohorts, had stroke patients with NIHSS < 5, received DAPT within 24 hours of stroke as intervention, and the comparator was thrombolysis within 4.5 hours of stroke. Additionally, studies published only in English were included. Studies were included if they documented at least one of the desired outcomes (mRS score, SICH, mortality).

Similarly, studies were excluded if they had stroke patients with NIHSS > 5 and received thrombolysis before DAPT or were given thrombolysis after 4.5 hours in the control group. Additionally, study designs other than RCTs and cohorts were excluded. Studies that did not report on any of the selected outcomes were also excluded.

### Screening, selection and quality assessment

Initially, the retrieved articles were searched for duplicates. After duplicate removal, a pair of reviewers separately reviewed the articles using their titles and abstracts. The remaining articles were searched for full text, and articles with available full text were screened against the prespecified eligibility criteria. In the event of any confusion regarding the inclusion or exclusion of an article, a third reviewer was consulted to resolve any disagreements.

The quality of the RCTs was assessed using the ROB2 tool, and that of the cohorts using the Newcastle-Ottawa Scale. ROB2 consists of 5 domains. If all domains demonstrate low risk of bias, the overall study is considered to have low risk of bias. A study is regarded as high risk if one domain shows high risk or two domains show some concerns. Similarly, some concerns regarding bias are assigned to a study with a single domain, while the remaining domains are considered to have a low risk of bias. For cohorts, the Newcastle-Ottawa scale was used, which consists of three domains and eight items. A study was assessed to be of poor quality if the score was below 5, good quality if the score was between 8 and 9 and fair quality if the score was between 6 and 7^9)^.

### Data extraction and synthesis

After screening, the included articles underwent data extraction for the following: author name, year, study design, sample size, male-to-female ratio, age, intervention, comparator, and outcomes analysed.

RevMan 5.4 was used to analyse the data extracted from the selected studies─a random-effects Mantel-Haenszel model was employed. For dichotomous variables, we used risk ratios rather than odds ratios, as suggested by the Cochrane Handbook^10)^. Similarly, as recommended by the Cochrane Handbook, RCTs and cohorts would be analyzed separately instead of combining them in a single meta-analysis because one should expect more heterogeneity in an observational study compared to an RCT due to the diversity in the methodology of both study designs. Forest plots were used to present the results of the analysis. Sensitivity analysis was also conducted to identify and rule out potential causes of heterogeneity. Heterogeneity was considered high if I2 was greater than 75%, moderate if 51-75%, mild if 26-50%, and low if it was below 25%. For all analyses, p < 0.05 was interpreted as significant.

## Results

A total of 3,355 articles were retrieved from our selected databases using the keywords mentioned above. Out of 3,355, 347 duplicates were removed, followed by the removal of 2,901 records based on their titles and abstracts. The full text of 14 articles couldn’t be found. Thus, the remaining 93 articles were assessed for eligibility, out of which only seven articles were selected for our review. 1 RCT and six cohorts were included in the list of articles included in the meta-analysis. As suggested by The Cochrane Handbook, combining RCTs and cohorts in a single analysis can lead to extreme heterogeneity, resulting in unreliable results. We removed the RCT from our review because we couldn't find more than one RCT on this topic for a separate analysis of RCTs^10)^. Thus, only six cohorts were included in our final analysis^11-16)^. The flowchart for screening and selection is given below in Figure 1

**Figure 1:**
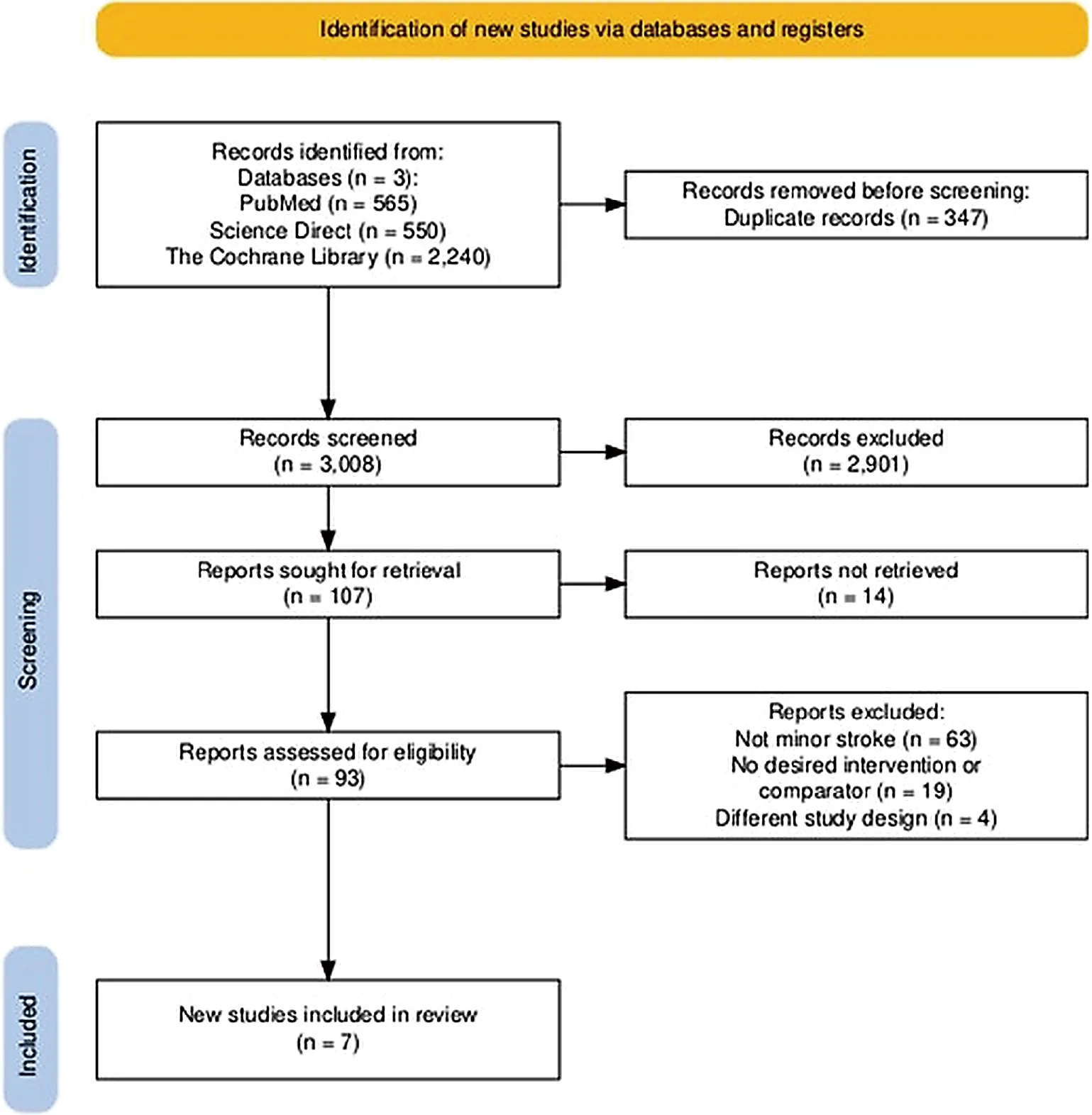
PRISMA flowchart for screening and selection of articles

### Study characteristics

Of the chosen studies, two were prospective, while 4 were retrospective cohorts. The overall sample size of this review was 6,402, comprising 4,054 participants in the DAPT group (63.3%) and 2,348 participants in the t-PA group (36.7%). Overall, males outnumbered females, with 4151 males compared to 2251 females. The age range was from 19 to 99 years. Aspirin and clopidogrel were used as DAPT, and alteplase was predominantly used for thrombolysis. The doses of aspirin and clopidogrel ranged from 75 to 300 mg, while t-PA was administered at a rate of 0.9 mg/kg. Further details are provided in Table 1 below.

**Table 1:** Characteristics of included studies

Study ID	Study design	Sample size (DAPT/IVT)	Males (DAPT/IVT)	Age (DAPT/IVT)	NIHSS (DAPT/IVT)	Intervention	Comparator	Outcomes
Duan et al., 2023	Prospective cohort	722/251	510/178	63(56-70)/ 62(55-68)	2.0(1-4)/ 3.0(2-4)	Aspirin 75 mg + Clopidogrel 75-300 mg followed by 75 mg	IV t-pa 0.9 mg/kg with a maximum dose of up to 90 mg	mRS scores, SICH, mortality
Lan et al., 2019	Retrospective cohort	119/109	86/74	64(32-86)/ 67(33-89)	2.5(0-5)/ 4(1-5)	Aspirin 75-300 mg followed by 75 mg Clopidogrel 300 mg followed by 75 mg	Alteplase 0.9 mg/kg	mRS scores, SICH
Sykora et al., 2023	Retrospective cohort	2,625/1,195	1,609/751	71(19-99)/ 68(21-98)	1(0,3-2)/ 2(0-3.2)	Aspirin 75-300 mg followed by 75 mg Clopidogrel 300 mg followed by 75 mg	IV t-pa 0.9 mg/kg	mRS scores, SICH
Wang et al., 2021	Retrospective cohort	215/385	149/257	64(55-71)/ 61(54-69)	2(1-3)/ 2(1-3)	Aspirin 75-300 mg followed by 75 mg Clopidogrel 300 mg followed by 75 mg	IV t-pa 0.9 mg/kg	mRS scores, SICH, mortality
Cui et al., 2024	Retrospective cohort	342/377	231/265	65 (57-71)/ 64 (56-71)	2(1-3)/ 2(1-3)	Aspirin 100 mg followed by 100 mg Clopidogrel 300 mg followed by 75 mg	Alteplase 0.9 mg/kg	mRS scores, SICH, mortality
Jiang et al., 2025	Prospective cohort	31/31	18/23	66(56-76)/ 65 (54-73)	1.0(1.0-2.0)/ 4.0(2.0-5.0)	-	-	mRS scores, SICH

### Quality assessment

Quality assessment using the NOS revealed that 4 out of 6 studies had fair quality, while only 2 had high quality, as shown in Table 2.

**Table 2:** Quality assessment of included studies using the Newcastle-Ottawa Scale (NOS)

Study (Author, Year)	Selection (0-4)	Comparability (0-2)	Outcome (0-3)	Total (0-9)	Quality
Sykora et al. (2023)	★★★	★★	★★	7	Fair
Wang et al. (2021)	★★	★★	★★★	7	fair
Duan et al. (2023)	★★★	★★	★★★	8	High
Cui et al. (2024)	★★	★★	★★★	7	fair
Jiang et al. (2025)	★★	★★	★★	6	fair
Lan et al. (2019)	★★★	★★	★★★	8	High

### mRS scores

The primary efficacy outcome was the mRS score of 0-1, as reported in all included studies. The meta-analysis of the risk ratios revealed no significant difference between DAPT and IVT in achieving an mRS score of 0-1 (RR, 1.03; 95% CI 0.98-1.09; p = 0.24), with moderate statistical heterogeneity observed (I2 = 68%). A forest plot depicting the results is given below in Figure 2.

**Figure 2:**
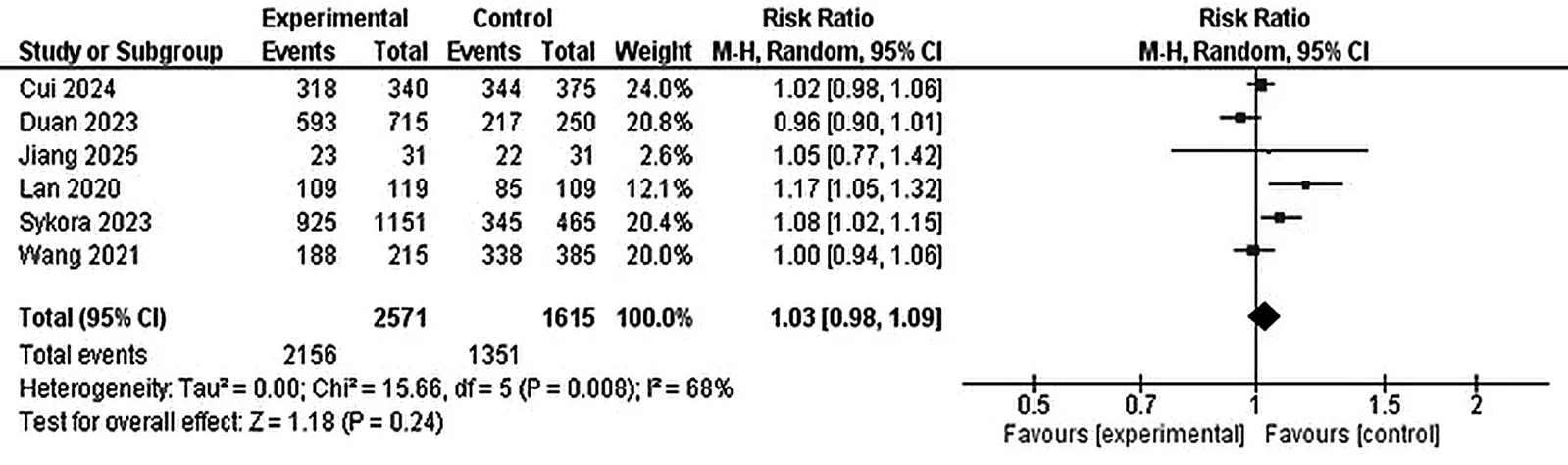
Forest plot for mRS score 0-1

Additionally, five studies reported on the mRS score of 0-2. However, as shown above, no difference was observed between DAPT and IVT in achieving an mRS score of 0-2 (RR 1.00; 95% CI 0.98-1.03; p = 0.87), and mild heterogeneity was observed (I2 = 30%). The forest plot is shown in Figure 3.

**Figure 3:**
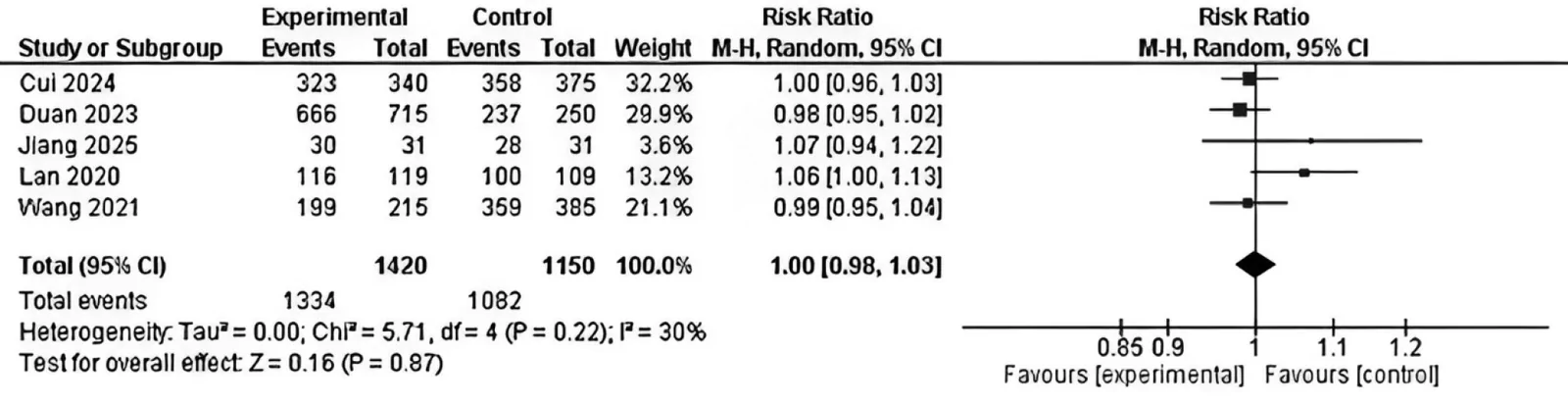
Forest plot for mRS 0-2

### SICH and mortality

To assess safety measures, three studies examined the risk of SICH. Results showed that the risk of SICH was significantly reduced with DAPT compared to IVT at 90 days (RR 0.10; 95% CI 0.03-0.29; p < 0.0001), with no statistical heterogeneity. A forest plot summarising the results is given below in Figure 4.

**Figure 4:**
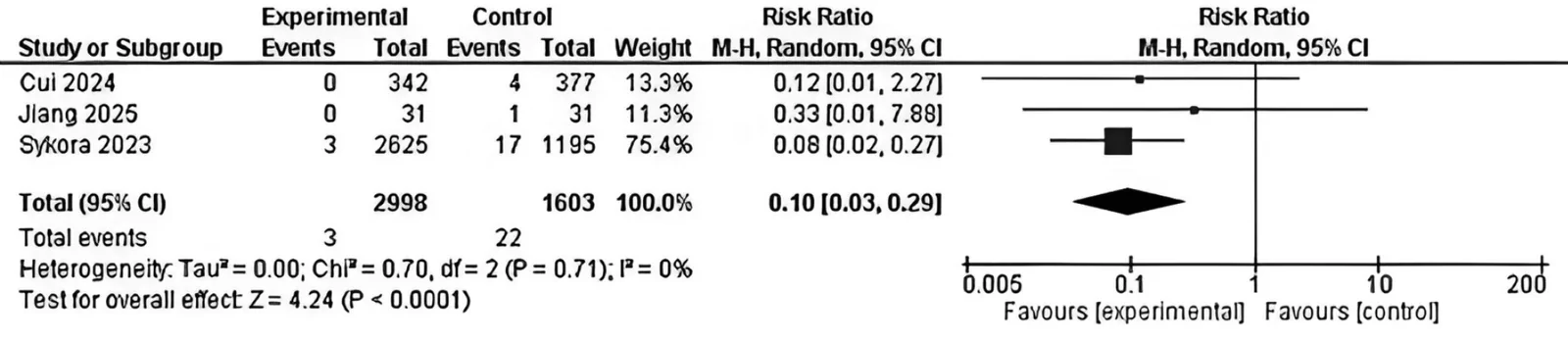
Forest plot showing risk of SICH

Similarly, three studies assessed mortality, and the results showed that mortality was reduced with DAPT compared to thrombolysis; however, no statistical significance was observed (RR 0.87; 95% CI 0.22-3.40; p = 0.84). No statistical heterogeneity was observed. The results for mortality are presented in Figure 5.

**Figure 5:**
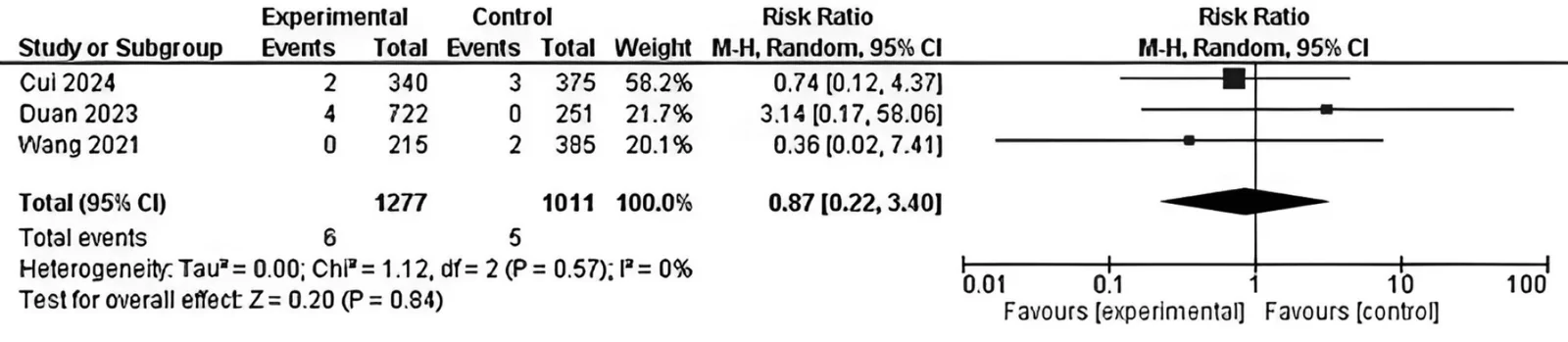
Forest plot showing the risk of death

### Sensitivity analysis

Due to the limited number of studies, a sensitivity analysis could only be conducted on efficacy measures. A leave-one-out sensitivity analysis did not alter the results for mRS scores 0-1, and statistical heterogeneity did not decrease either.

For the mRS score of 0-2, although the results did not change, the heterogeneity decreased to 0 after the removal of the study by Lan et al.^12)^.

## Discussion

This meta-analysis evaluated both the efficacy and safety of DAPT and IVT in patients presenting with minor ischemic stroke (NIHSS score ≤ 5). The results indicate that, while no meaningful statistical difference in functional outcomes (modified Rankin Scale [mRS] scores 0-1 and 0-2) was observed between the two treatment strategies at 90 days, DAPT is associated with a significantly lower risk of SICH. Mortality rates were also lower with DAPT, though this difference was not statistically significant.

Our outcomes, indicating that IVT and DAPT result in comparable functional recovery, align with an increasing body of evidence questioning the overall effectiveness of thrombolysis in patients with mild, non-disabling ischemic stroke. Our evaluation is directly informed by the landmark PRISMS study, which demonstrated no significant variation between the alteplase and aspirin in patient distribution across groups who achieved an mRS of 0-1 at 90 days (RR 1.08; p = 0.45)^17)^. These findings contrast with earlier thrombolysis trials such as NINDS and ECASS III, which established IVT efficacy across broader stroke populations but were not sufficiently powered to evaluate minor stroke subgroups specifically^18, 19)^. Collectively, these data suggest that intensive antiplatelet therapy may be sufficient to promote functional independence in many patients with minor stroke, particularly in the absence of large vessel occlusion (LVO)^20, 21)^.This contradicts the conventional paradigm based on larger thrombolysis trials, such as NINDS and ECASS III, which established the effectiveness of IVT but did not have sufficient power for the mild stroke subgroup and covered strokes of all severities^18, 19)^. According to our findings, vigorous antiplatelet therapy may be just as successful in fostering functional independence in a substantial number of patients with mild stroke, especially those without established large vascular occlusion (LVO)^20, 21)^.

Furthermore, our findings are further supported by the minimal variability and stability of the mRS 0-2 result across investigations. IVT was not associated with better functional outcomes in small strokes compared with antiplatelet treatment alone, according to findings from the ARAMIS registry analysis and a large comparative effectiveness investigation using US registry data^22, 23)^. Sensitivity analyses in our study showed that exclusion of a single study eliminated heterogeneity for mRS 0-2, suggesting that patient selection criteria or protocol-specific factors may influence observed effects,In our sensitivity analysis for mRS 0-2, the elimination of a single study to bring heterogeneity to zero raises the possibility that patient selection or particular protocols in that study were an anomaly, supporting the general finding that there is no difference when conventional procedures are followed^24, 25)^. Moreover, our analysis supports this hypothesis by integrating more recent, focused comparisons of DAPT and IVT. Given the similar effectiveness, strong antiplatelet medication may be enough to help many patients with mild stroke recover well without having to undergo thrombolysis. The CHANCE and POINT trials demonstrated the effectiveness of DAPT in reducing early stroke recurrence in small strokes and TIA, lending more credence to this^26)^.

Additionally, one significant finding is that the mRS 0-1 result exhibits moderate heterogeneity (I2 = 68%). Potential clinical sources of heterogeneity include differences in baseline NIHSS scores, prevalence of LVO, imaging-based patient selection, stroke etiology, and treatment timing across studies. Variations in patient demographics, such as the frequency of large vessel occlusion (LVO), may be responsible for this fluctuation. Research on minor stroke patients with LVO, such as that by Duan et al. (2023)^27)^, which was part of this analysis, may demonstrate a different therapeutic impact than studies of minor strokes that were not chosen. Some observational studies support the theory that thrombolysis is more effective for people with LVO. The persistence of heterogeneity despite sensitivity analyses suggests that unmeasured clinical factors likely contribute to outcome variability, The differing findings among trials may be caused by other unmeasured factors, such as time to treatment or a particular stroke etiology, according to the sensitivity analysis, which did not reduce heterogeneity^26)^.

However, DAPT significantly reduces the risk of SICH, which is the analysis's most notable conclusion. While antiplatelet medications primarily prevent the formation of new thrombi, IV thrombolytic agents are designed to break down clots actively, carrying an intrinsic risk of hemorrhagic transformation^25, 26)^. This is entirely consistent with the known pharmacological characteristics of both therapies. The very low rates (often less than 1%) documented in trials such as CHANCE and POINT, which demonstrated the safety of DAPT in mild stroke, are consistent with the SICH rate in our DAPT group^28, 29)^. The SICH rate in the IVT arm, on the other hand, is consistent with the ~2-3% risk pool identified by SITS-MOST and IST-3, two large thrombolysis registries and studies^30-32)^. Notably, clinical decision- making heavily depends on this safety signal. Although there is little chance of SICH with IVT, the effects can be deadly and severe. Our findings confirm concerns raised in analyses of the PRISMS and IST-3 trials, which found that thrombolysis increased the risk of early fatal bleeding in milder strokes, thereby negating its benefits in this particular cohort^17, 33, 34)^. Thus, when efficacy is equal, DAPT offers a substantial clinical benefit by avoiding this risk.

Additionally, because fatal SICH events are avoided, DAPT has been shown to reduce mortality in a non-significant way. This result aligns with the CHANCE trial, which showed that DAPT reduced mortality, albeit non-significantly, compared to aspirin alone^28, 35)^. In minor stroke, where baseline mortality risk is low, safety and functional independence may be more clinically meaningful endpoints than survival alone. Larger meta-analyses of thrombolysis have revealed a minor or neutral effect on death at 90 days, with the primary objective being the reduction of disability^36, 37)^. According to our data and those of others, neither DAPT nor IVT significantly affects mortality in the setting of mild stroke, when the baseline risk of death is minimal; instead, safety and functional outcomes are the more critical differentiators^36, 37)^.

### Limitations and Strengths

Our meta-analysis is limited in several ways. The first factor that could affect the accuracy of our estimates is the relatively small number of studies included for the safety outcomes (SICH and mortality). A key limitation is the inclusion of both randomized controlled trials (RCTs) and observational cohort studies. While this provides a broader evidence base, it introduces a potential for bias, as cohort studies may be more susceptible to confounding by indication and other unmeasured variables compared to RCTs. The presence of moderate statistical heterogeneity for the primary efficacy outcome (mRS 0-1) indicates that, even with the sensitivity analysis, our analysis was unable to fully account for clinical or methodological differences among the included trials (e.g., differences in DAPT regimens, time-to-treatment windows, or definitions of “minor stroke"). Finally, because this was a study-level meta-analysis, we were unable to conduct patient-level subgroup analyses to determine which specific patient features might alter the effects of treatment. Despite these limitations, the review possesses notable strengths. It adhered to rigorous systematic review and meta- analysis methodology. It focused on a highly relevant and contemporary clinical question and synthesised data from the most recent direct-comparison trials. The use of sensitivity analyses strengthens the credibility of the findings, and the results for both efficacy and safety are highly consistent with the known pathophysiology and pharmacology of the treatments being compared. From a generalizability perspective, the predominance of cohort data in this analysis reflects real-world clinical practice. These findings may be particularly relevant in healthcare settings with limited access to thrombolysis, advanced imaging, or specialized stroke centers, where DAPT represents a readily available, lower-risk therapeutic option.

## Conclusion

Among patients experiencing minor ischemic stroke (NIHSS score ≤ 5), both IVT and DAPT provide comparable functional status at 90 days, showing no meaningful difference in mRS scores. However, DAPT correlates with a significantly lower risk of SICH, supporting its safety profile. While mortality rates were lower with DAPT, this difference did not reach statistical significance, aligning with findings from previous large trials. Our analysis underscores growing evidence that thrombolysis may not offer superior functional recovery for mild stroke patients, particularly those without large vessel occlusion (LVO). The findings of our study, combined with the PRISMS, CHANCE, and POINT trials, suggest that strong antiplatelet therapy could be sufficient for promoting recovery in many cases of minor ischemic stroke. Our analysis underscores growing evidence that thrombolysis may not offer superior functional recovery for mild stroke patients, particularly those without large vessel occlusion (LVO). Importantly, DAPT's reduced risk of SICH and its potential to lower mortality offer a substantial safety advantage, further supporting its use as a first-line treatment option in mild stroke, particularly in settings where thrombolysis may pose unnecessary risks.

## Availability of data and materials

All data used in this study are publicly available from the original published articles cited in the references.

## Author contributions

Conceptualization: MS, HA; Data curation: AJC, AR, WD; Formal analysis: RZ, ZH; Investigation: MSH, AF; Methodology: SHFS, AI; Project administration: MS; Resources: HA; Software: AJC; Supervision: MS, HA; Validation: ZH; Visualization: ZS, WD; Writing-original draft: RZ; Writing-review & editing: all authors.

## Conflicts of interest statement

The authors declare that there are no conflicts of interest.
